# BRCA1 and Breast Cancer: Molecular Mechanisms and Therapeutic Strategies

**DOI:** 10.3389/fcell.2022.813457

**Published:** 2022-03-01

**Authors:** Xiaoyu Fu, Wei Tan, Qibin Song, Huadong Pei, Juanjuan Li

**Affiliations:** ^1^ Department of Breast and Thyroid Surgery, Renmin Hospital of Wuhan University, Wuhan, China; ^2^ Cancer Center, Renmin Hospital of Wuhan University, Wuhan, China; ^3^ Department of Biochemistry and Molecular Medicine, The George Washington University School of Medicine and Health Sciences, Washington, DC, United States; ^4^ Department of Oncology, Georgetown Lombardi Comprehensive Cancer Center, Georgetown University Medical Center, Washington, DC, United States

**Keywords:** BRCA1, gene mutation, breast cancer, detection, personalized and precision medicine (PPM)

## Abstract

Breast cancer susceptibility gene 1 (*BRCA1*) is a tumor suppressor gene, which is mainly involved in the repair of DNA damage, cell cycle regulation, maintenance of genome stability, and other important physiological processes. Mutations or defects in the *BRCA1* gene significantly increase the risk of breast, ovarian, prostate, and other cancers in carriers. In this review, we summarized the molecular functions and regulation of *BRCA1* and discussed recent insights into the detection and treatment of *BRCA1* mutated breast cancer.

## Introduction

Breast cancer (BC) is the most common malignancy all over the world, accounting for 11.7% of new cancer cases (Sung et al., 2021). Up to 7% of unselected BC patients have a definite germline genetic mutation called hereditary breast cancer (HBC) (Claus et al., 1996). Among them, breast cancer susceptibility gene 1 (*BRCA1*) is one of the most common tumor suppressor genes, which encodes a 220 kD nuclear protein and is detected in at least 5% of unselected patients with BC (Hall et al., 1990; Chen et al., 1996). *BRCA1* plays an important role in DNA repair, replication fork protection, cell cycle regulation, and gene transcription regulation (Gudmundsdottir and Ashworth, 2006). When the *BRCA1* gene is mutated or lost, the incidence of BC and ovarian cancer will increase significantly (Miki, et al., 1994). The cumulative risk of BC by 80 years of age in healthy female carriers of *BRCA1* mutation is about 80% (Kuchenbaecker et al., 2017; King et al., 2003) while one in eight women will develop BC over the lifespan in the general population. Carriers of *BRCA1* mutation are more likely to develop triple-negative breast cancer (TNBC), which suggests that *BRCA1* mutation and the hormone receptor status are interlinked (Foulkes et al., 2004). *BRCA1*-mutated BC is associated with earlier onset, more aggressive disease, and a higher risk of relapse. Hence, it is important to investigate the function and dysregulation of *BRCA1* in BC and treatment strategies for this population. In this article, we provide a comprehensive overview of *BRCA1* in BC, including the *BRCA1’s* molecular function, its mutation detection, and the prevention and treatment of BC in mutated carriers.

## *BRCA1* Gene

### Structure and Function of *BRCA1* Gene

**FIGURE 1:**
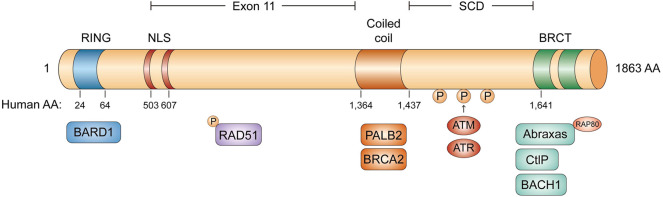
The domain structure of BRCA1. The RING domain in blue, the two NLS domain in red, the coiled coil domain in orange, and the two BRCT domains in green. BRCA1 can form four different complexes: BRCA1/RAP80/Abraxas complex, BRCA1/BACH1 complex, BRCA1/PALB2/BRCA2 complex and BRCA1/CtIP complex.

*BRCA1* is an incomplete recessive gene on an autosome, located on chromosome 17q21 and encoded 220 kD protein-containing multi-function domains (Hall et al., 1990). There are 24 exons in *BRCA1* whose exons 2–5 encode the RING domain and exons 15–23 encode the *BRCA1* C-terminal (BRCT) domain (Figure 1) (Miki et al., 1994; Clark et al., 2012). The N-terminal RING domain has an E3 ligase activity, which interacts with its partner protein, the *BRCA1*-associated RING domain protein 1 (BARD1) to form a stable BRCA1-BARD1 heterodimer (Hashizume et al., 2001). The BRCT domain is associated with different phosphorylated interacting proteins. In addition to the N-terminal RING domain and C-terminal domain, there is a coiled-coil domain upstream of BRCT domains, which binds another coiled-coil domain at the N-terminus of PALB2. PALB2 also binds BRCA2 and serves as the molecular scaffold in the formation of the BRCA1-PALB2-BRCA2 complex (Shirley et al., 2009; Zhang et al., 2009). In mammalian cells, homologous recombination (HR) and non-homologous end-joining (NHEJ) are two major repair pathways to repair DNA double-strand breaks (DSBs) for genome integrity. Both the RING domain and BRCT domain of *BRCA1* are essential for HR to maintain genome stability. Many clinically important mutations of *BRCA1* gene frequently target these two domains.

**FIGURE 2:**
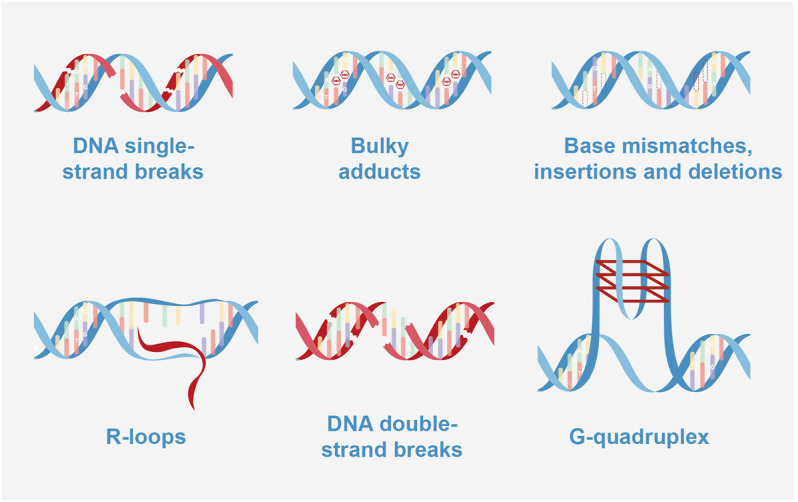
Types of DNA damage. DNA single-strand breaks: BER (base excision repair); bulky adducts: NER (nucleotide excision repair); base mismatches, insertions and deletions: MMR (Mismatch repair); R-loops caused double strand breaks (DSB): NHEJ (Non-homologous end joining) and HRR (homologous recombination repair); DNA double-strand breaks: NHEJ and HRR; G-quadruplex caused DSB: NHEJ, and HRR.

The BRCT domain is conserved in several DNA damage response (DDR) proteins and is responsible for *BRCA1* to recognize a phospho-SPxF motif (S, serine; P, proline; x, varies; F, phenylalanine) (Wang, 2012; Chabanon et al., 2021). Figure 2 BRCA1 can form four different complexes in cells, through the association of different adaptor proteins with the BRCT domain, such as BRCA1/RAP80/Abraxas complex, BRCA1/BACH1(*BRCA1* associated C-terminal helicase) complex, BRCA1/PALB2 (partner and localizer of *BRCA2*)*/*BRCA2 complex, and BRCA1/CtIP complex (Figure 1). BRCA1/RAP80/Abraxas complex is recruited to DNA DSBs through RAP80, a ubiquitin-binding protein. RAP80 could target this complex to MDC1-rH2AX-dependent K6 and K63-linked ubiquitin polymers at DSBs. BRCA1/RAP80/Abraxas complex prevents excessive end resection and potentially deleterious homology-directed DSB repair mechanisms (Huang and Zhou, 2020; Vohhodina et al., 2020). The helicase catalytic function of BRCA1/BACH1 is not only important for BRCA1-mediated DDR but also necessarily required to maintain DNA damage-induced G2/M checkpoint (Yu et al., 2003). As described previously, PALB2, the partner and localizer of BRCA2, could bind directly to BRCA1 to form BRCA1/PALB2/BRCA2 complex, which stimulates RAD51-mediated localization and repair at DNA breaks (Shirley et al., 2009; Ducy et al., 2019). Lastly, BRCA1/CtIP complex promotes the HR by DNA end resection.

In addition to its critical roles in DSB repair, *BRCA1* is also involved in the repair and restart of stalled and damaged DNA replication forks and in the protection from nucleolytic attack and degradation. BRCA1-mediated fork protection functions independently from its role in the HR-mediated repair of DNA DSBs (Schlacher et al., 2012; Long et al., 2014). Upon replication fork stress, BRCA1 protects nascent DNA strands from degradation by stabilizing *RAD51* nucleofilaments that affect the exonuclease activity of *MRE11* (Schlacher et al., 2012). *RAD51* is also required for fork restart once halted forks are repaired in response to short replication blocks (Petermann et al., 2010). Moreover, BRCA1 also has important roles in gene transcription. Gerald M. Pao et al*.* have shown that the carboxyl terminus of *BRCA1* transactivates the heterologous promoters (Pao et al., 2000). This BRCA1-mediated transactivation could be mediated by RNA polymerase II (Pol II) *via* RNA helicase A (RHA) and enhanced by transcriptional coactivators/acetyltransferases p300 and CBP (p300/CBP). Zhu et al. also showed that BRCA1 could bind to satellite DNA regions and ubiquitylates the histone H2A to maintain the heterochromatin structures (Zhu et al., 2011; Zhu et al., 2018); Bochar et al. (Bochar et al., 2000) reported that BRCA1 is a component of SWI/SNF chromatin remodeling complex and controls the transcription through the modulation of chromatin structure. RNA/DNA hybrid structures (R-loops) as normal transcriptional intermediates also affect transcription and genomic instability. BRCA1 is recruited to transcriptional pause sites and mediates the recruitment of senataxin (SETX) (Hatchi et al., 2015); SETX is involved in processing replication forks and resolves R-loops at transcriptional sites; thus, BRCA1/SETX addresses R-loop associated DNA damage arising at transcriptional pause sites. Recently, other studies showed that BRCA1 aberrantly retains at the transcription regions with increasing R-loop levels and decreases its distribution at DNA damage regions in Ewing sarcoma cells (Gorthi et al., 2018). As a result, these cells could not inhibit the transcription after DNA damage and showed a defect in HR repair. Furthermore, Steffi Herold et al. found more details about the relationship between BRCA1, R-loop, and transcriptional regulation in human neuroblastoma cells (Herold et al., 2019). In the MYCN-amplified cells, MYCN activation could increase the transcriptional elongation by inducing the escape of RNAPII from promoters. The recruitment of BRCA1 to the promoter-proximal regions could stabilize MYCN on the chromatin and prevent R-loop formation caused by the RNAPII stalling at the transcription-suspended sites.

### The Mutation of *BRCA1* and Breast Cancer

Since the first clone of the *BRCA1* gene in 1994 (Miki et al., 1994), variable cut transcripts were found as a “naturally occurring” event in both tumor and normal tissues by many studies (Li et al., 2019). There are at least six alternative splicing transcripts of *BRCA1* discovered, including *BRCA1* exon 1a, exon 1b, exon 1c, *BRCA1*a (∆11q, ∆11), *BRCA1*b (∆9,10), and *BRCA1-IRIS*, which codes protein products with different molecular weights. Based on these observations, Walker et al. revised 77 published studies with 252 BRCA1 splicing analysis assays and found that some of the exon boundary variants may not perform as a loss of function, leading to a naturally occurring in-frame RNA isoform (Walker et al., 2013). Compared to the complete transcript of *BRCA1*, the variable-cut transcripts of *BRCA1* can have similar or opposite functions and, in some cases, may have more unique functions (Braunschweig et al., 2013). Up to now, 1,800 mutations have been found in human *BRCA1*, including intron mutations, missense mutations, nonsense mutations, frameshift mutations, and other types. These mutations often occur in the RING and BRCT domains, which are the key domains in *BRCA1* for genome integrity (Supplementary Data Sheet). Missense mutations in *BRCA1* present a significant challenge for the prevention and treatment of patients. For example, *BRCA1* c.5309G > T p. (Gly1770Val) has been shown to inhibit homologous recombination and could be considered as a disease-causing mutation (Tudini et al., 2018). As a benefit from bioinformatic analysis, more variants of *BRCA1* can be found from public databases, such as the cBioPortal database (http://www.cbioportal.org/), ENIGMA (https://enigmaconsortium.org/), BRCA Exchange (https://brcaexchange.org), and ClinVar (https://www.ncbi.nlm.nih.gov/clinvar/).

Patients with *BRCA1* mutations have a higher risk for cancer. The estimated lifetime risk of BC is about 80%, and the lifetime risk of ovarian cancer is 40%–65% (King et al., 2003; Kuchenbaecker et al., 2017), which might alter according to the type and location of the mutations (Rebbeck, et al., 2015). *BRCA1* gene deletion with or without p53 defect leads to a high incidence of basal-like BC and tends to form TNBC, which is the most aggressive type of BC (Tarsounas and Sung, 2020). Some studies show that the TNBC in *BRCA1* mutation carriers originated from luminal progenitor cells, not basal stem cells (Lim et al., 2009; Molyneux et al., 2010). If *BRCA1/p53* is perturbed in luminal progenitors, it could induce the abnormal alveolar differentiation premalignancy with pro-tumorigenic changes in the immune compartment. It belongs to cell autonomy and is caused by the dysregulation of transcription factors. This study explains how *BRCA1* aberration impacts the state of nascent tumor cells and their microenvironment. Bach et al. found that breast cells with *BRCA1* mutations undergo changes similar to those common changes in women during pregnancy (Bach et al., 2021). Based on the data, they proposed a model in which *BRCA1/p53*-driven transcriptional and epigenetic changes inadvertently promote innate differentiation programs in luminal progenitors accompanied by protumorigenic changes in the immune compartment, highlighting the decisive role of the origin cell and providing a potential explanation for the tissue specificity of *BRCA1* tumors. Researchers have mapped early changes in seemingly healthy breast tissue before tumors appear, which may have great significance for the early diagnosis of BC (Bach et al., 2021).

In addition to familial BC, *BRCA1* gene silencing due to promoter methylation can also lead to sporadic BC (Esteller et al., 2000). A study of tumor xenografts from TNBC patients (Ter Brugge et al., 2016) revealed a novel resistance mechanism in *BRCA1*-methylated PDX (patient-derived xenograft) tumors. Next-generation sequencing (NGS) data showed that the genome rearrangement places the *BRCA1* gene under the transcriptional control of the heterologous promoter, which results in the re-expression of *BRCA1* in a subset of *BRCA1*-mutated PDX tumors and leads to acquired resistance to PARP [poly (ADP-ribose) polymerase 1,2] inhibitor (PARPi) and cisplatin chemotherapy (Ter Brugge et al., 2016). This is a unique example of genomic plasticity that is caused by the treatment of *BRCA1*-deficient tumors, but it can lead to tumor regeneration.

## Detection of *BRCA1* Gene Mutations

The *BRCA1* genetic test is designed to identify harmful changes in *BRCA1* using a blood test. People who inherit mutation in *BRCA1* gene are at an increased risk of developing BC and ovarian cancer than the general population. Therefore, the *BRCA1* gene test has been widely used by physicians to develop risk-reducing strategies for those who are likely to have an inherited mutation based on personal or family history. Additionally, *BRCA1* mutation is a prognostic and predictive biomarker for BC. Although studies provided conflicting interpretations of the prognostic value of *BRCA1* mutation in BC patients (Lee et al., 2010; Copson et al., 2018), patients with *BRCA1* mutation may be sensitive to platinum salts and PARP inhibitors, which could significantly prolong survival time. Hence, *BRCA1* genetic testing is essential for making individualized therapy for selected BC patients. According to the consensus of experts and guidelines, the criteria for candidates to do *BRCA1* genetic testing are (Daly et al., 2021): 1) BC patients before the age of 40 years; 2) BC patients before the age of 50 years old who had a second primary BC or a history of BC or pancreatic or prostate cancer in their relatives; 3) patients with TNBC before the age of 60 years old; 4) all male BC patients; 5) patients with bilateral BC; and 6) Patients with a relevant family history at any age, who want to be assessed for cancer risk.

The common gene detection methods for *BRCA1* mutations include Sanger sequencing, NGS, multiplex ligation-dependent probe amplification (MLPA), massively parallel signature sequencing (MPSS), chromosomal microarray (CMA), and array comparative genomic hybridization (aCGH). (Toland et al., 2018). So far, no single technique can detect all mutations in the *BRCA1* gene (Walsh et al., 2010).

In order to reduce the rate of missed detection, a combination of several methods is used to detect all mutations. Previously, *BRCA1* gene detection is mainly performed by Sanger sequencing and MLPA, which can screen out single-nucleotide mutation, small fragment mutation, and large copy number mutation (CNVs). Nowadays, NGS is generally used for gene detection as a high-throughput gene sequencing technology (Plagnol et al., 2012). After the genomic DNA has been cut into small fragments, the end of the molecule is connected to the sequencing preparation library and the sequencing results are obtained after image collection and analysis (Gao and Smith, 2020). Compared with the previous gold standard Sanger sequencing, NGS is both cost and time effective, high throughput with simple operation, and operatable in clinical practice. The disadvantage is the increased error rate by introducing polymerase chain reaction in sequencing (Suryavanshi et al., 2017). For the detection of *BRCA1* gene mutations in the real world, there are two major patterns. It is recommended to use NGS technology combined with large fragment deletion detection to detect all exons of *BRCA* gene and the junction region between exons and introns ±20 bp to explore *BRCA1* gene mutation. If the mutation in the allele is identified from the proband in the family, it is appropriate to validate specific loci in the family using the Sanger sequencing method.

Most of the pathogenic mutations of *BRCA1* are frameshift mutations and nonsense mutations caused by a single or several base changes. A large fragment rearrangement variant should be considered when no mutations are found by conventional gene sequencing in HBC/hereditary ovarian cancer families (Schmidt et al., 2017). MLPA is the most commonly used method to detect large fragment rearrangement in *BRCA1* (Engert et al., 2008). When the polymorphic changes in the DNA of the binding site of the primers affect the binding force between the primers and the target fragment (allelic dropouts), it may lead to both false-positive and false-negative results. When a rearrangement variant is detected by an amplicon-based NGS panel, an additional MLPA assay may be considered for validation (Gomez et al., 2009). The targeting RNA-seq is used to analyze the naturally occurring splicing events of eight BC and/or ovarian cancer susceptibility genes (*BRCA1, BRCA2, RAD51C, Rad51d, PTEN, STK11, CDH1, TP53*). The results showed that the targeted RNA-seq could identify abnormal splicing events associated with *BRCA1* genetic variation and successfully distinguish between complete and incomplete splicing events, which is of great significance in determining pathogenicity (Brandao et al., 2019).

## Prevention Strategies in *BRCA1* Mutation Carriers

Female carriers of a *BRCA1* mutation face a higher lifetime risk to develop BC and ovarian cancer. In general, there are three risk-reducing strategies that have been recommended for these carriers: surveillance, risk-reducing surgery, and chemoprevention. Due to comprehensive considerations, the individual risk-reducing strategy should be discussed and made in terms of several factors including the risk from the specific mutation loci, age, general health status, and the life expectancy of the patient. This risk-reducing therapy should be discussed in a shared decision-making environment with a multidisciplinary team.

Until now, risk-reducing surgeries, a risk-reducing mastectomy (RRM) and risk-reducing salpingo-oophorectomy (RRSO), or a combination of both, have been considered to be most effective in preventing the onset of BC and ovarian cancer. RRM can reduce the risk of developing BC in *BRCA1* carriers by more than 90% (Meijers-Heijboer et al., 2001; Heemskerk-Gerritsen et al., 2013; Li et al., 2016) and even reduce mortality from any cause (Honold and Camus, 2018). The psychosocial effect after RRM should not be ignored, with respect to negative impacts on body image and sexuality. Thus, the NCCN guidelines recommend that women with a *BRCA1* mutation may undergo RRM with immediate bilateral breast reconstruction and multidisciplinary consultations before making treatment plans, and postoperative psychological counseling is necessary. The European Society of Medical Oncology (ESMO) (Cardoso et al., 2019) and NCCN (Esteller et al., 2000) of the United States stated that prophylactic RRSO may significantly reduce the risk of BC and ovarian cancer in women with *BRCA1* gene mutation after the completion of reproductive needs. Several prospective clinical trials demonstrated the efficacy of selective estrogen receptor modulators (Cuzick et al., 2013) (i.e., tamoxifen, raloxifene) and aromatase inhibitors exemestane (Goss et al., 2011) and anastrozole (Cuzick et al., 2014) for preventing BC in unselected women. However, there is limited evidence supporting the efficacy of those risk-reducing endocrine therapy options for the carriers of *BRCA1* mutations. Studies failed to show the efficacy of tamoxifen (King et al., 2001) or letrozole (Pujol et al., 2020) on decreasing BC incidence in women with germline *BRCA1* mutations.

## Treatment of Breast Cancer With *BRCA1* Mutation: Current Practice and Future Directions

In general, the most common treatments for BC include a combination of surgery, radiation, chemotherapy, hormone therapy, targeted therapy, and immunotherapy. The commonly used surgical treatment methods include breast-conserving surgery (BCS), and mastectomy with the option of breast reconstruction. However, whether BCS is oncologically safe for *BRCA* mutation carriers has remained controversial (Cao et al., 2019; Davey et al., 2021). For *BRCA1* mutation carriers, breast conservation, comprising of lumpectomy followed by whole breast radiation, was associated with higher local recurrence risk for BC patients with *BRCA1* mutation; however, *BRCA1* mutation was not associated with inferior survival outcomes. Since there is no prospective randomized controlled trial that directly compared BCS and ipsilateral mastectomy for BC patients with *BRCA1* mutation, it should be careful to consider BCS (Cao et al., 2019; Davey et al., 2021). Regarding the significant increased risk of developing contralateral BC (Mavaddat et al., 2013), for BC patients with mutations in *BRCA1*, bilateral nipple-sparing mastectomy combined with reconstruction is a reasonable option for *BRCA1* mutation carriers (Tung et al., 2020a).

### PARP Inhibitor and Metastatic Breast Cancer With *BRCA1* Mutations

PARP inhibitions could induce the death of *BRCA1*-deficient cells and tumors by interfering with DNA replication, playing a synthetic lethal effect (Farmer et al., 2005; Lord and Ashworth, 2017). Olaparib and talazoparib, as PARP inhibitors, could bring improvement in progression-free survival (PFS) and tumor response rates, and likely improve overall survival for metastatic germline *BRCA1-*mutated, HER2-negative BC patients based on the primary results from two phase III randomized controlled trials (OlympiAD (Robson et al., 2019) and EMBRACA (Litton et al., 2020)), respectively. Thus, they received approval from the US Food and Drug Administration (FDA) and the European Medicines Agency (EMA). More recently, olaparib monotherapy (Gelmon et al., 2021) and pamiparib (Sun et al., 2021), also showed a promising response in patients with advanced HER2-negative BC with a germline *BRCA1* mutation. Additionally, veliparib (Somlo et al., 2017; Dieras et al., 2020; Xu et al., 2021), and niraparib (Turner et al., 2021) are also in investigation for *BRCA1*-mutated metastatic BC with modest benefit for patients. Those data revealed that the actions of different inhibitors as a PARPi are not the same. Therefore, based on the results above and studies currently under way, PARPi remain a very active area of investigation for BC with *BRCA1* mutation.

Based on preliminary data, there is an ongoing trial to explore the efficacy and safety of PARP inhibitors combined with apatinib (NCT04296370). Ceralasertib, an ataxia telangiectasia and Rad3-related protein ATR inhibitor, targets DNA damage repair and cell cycle regulation and shows synergistic antitumor effects combined with olaparib in preclinical studies and a pilot clinical trial (Mahdi et al., 2021).

### PARP Inhibitor and Early Breast Cancer With *BRCA1* Mutations

In the neoadjuvant setting, a series of clinical trials explored the role of PARP inhibitors; however, the data cannot achieve a clear conclusion. In the BrightNess trial, with the patients with germline *BRCA* mutation, the pathological complete response (pCR) rate was 57% in the veliparib combined with the carboplatin/paclitaxel arm, 50% in the placebo-carboplatin/paclitaxel arm, and 41% in the control paclitaxel arm (Loibl et al., 2018a). The difference for adding veliparib was not significant, but the trial was not powered to detect it. In GeparOLA (Fasching et al., 2021), the olaparib-containing arm failed to reach its primary endpoint and had a similar pCR compared to the carboplatin-based arm (55.1% vs. 48.6%) in HER2-negative patients with germline *BRCA1/2* mutation, or somatic *BRCA1/2* mutation, or a high homologous recombination deficiency score. Of note, neoadjuvant single-agent talazoparib without chemotherapy showed promising antitumor activity with manageable toxicity (Litton et al., 2021) in *BRCA1*-mutated TNBC, which is close to standard chemotherapy in such patients based on previous studies.

In the multicentric, randomized, double-blinded, placebo-controlled phase III OlympiA trial (Tutt et al., 2021), adjuvant olaparib significantly reduced the risk of invasive disease recurrence or death by 42% in high-risk HER2-negative early BC with germline *BRCA1/2* mutations. Subgroup analysis showed a consistent benefit in patients with *BRCA1* mutations. Importantly, the rate of central nervous system (CNS) recurrence was lower with olaparib treatment than that of placebo arm, which suggests the action of olaparib. FDA-approved adjuvant olaparib for early HER2-negative BC patients with high-risk-carrying germline *BRCA1/2* mutation. The definition of high risk in BC patients was defined in Table 1. OlympiA (Tutt et al., 2021) emphasizes the need to conduct the *BRCA* genetic test early to allow individualized treatment, which would maximize long-term outcomes for the BC patients with germline *BRCA* mutations.

**TABLE 1:** Definition of high-risk population in OlympiA.

	Regimens	TNBC	HR+/HER2-
Patients receiving neoadjuvant chemotherapy	≥6 cycles neoadjuvant chemotherapy (anthracycline ± taxane) Neoadjuvant platinum is allowed, and no adjuvant chemotherapy for patients with residual disease	Non-pCR	Non-pCR and CPS + EG score ≥3
Patients with initial surgery	≥6 cycles adjuvant chemotherapy (adjuvant platinum is allowed).	≥pT2 or ≥ pN1, any T	≥pN2 (at least four positive lymph nodes)
•Initially designed to enroll only TNBC patients in Apr. 2014. hen, the protocol was amended to include HR-positive, HER2-negative breast cancer patients, in Nov. 2015.			

Abbreviations: pCR, pathological complete response; HR, Hormone receptor; HER2, human epidermal growth factor receptor 2; CPS+EG, clinical-pathologic staging system that incorporates ER status and nuclear grading.

### Platinum Salts and Early Breast Cancer With *BRCA1* Mutations

Retrospective clinical studies in the neoadjuvant setting have shown that early BC patients with *BRCA1* mutations are more sensitive to platinum salts (Byrski et al., 2010; Arun et al., 2011; Saether et al., 2018; Holanek et al., 2019). However, the role of neoadjuvant platinum in patients with *BRCA1* mutation is still unclear due to the conflicting data from several prospective clinical trials. GeparSixto (Hahnen et al., 2017; Loibl et al., 2018b) and CALGB 40603 (Sikov et al., 2015) trials demonstrated that early TNBC patients benefit from platinum-based neoadjuvant chemotherapy with higher pCR rates than that from platinum-free neoadjuvant chemotherapy. However, results of the *post-hoc* exploratory subgroup analyses from GeparSixto failed to demonstrate that *BRCA1* mutation could predict higher pathological response. Furthermore, the addition of platinum could yield comparably high pCR for *BRCA1* mutation and wild-type patients (Sharma et al., 2017). Nevertheless, pCR tended to be worse in the cisplatin- containing group than in the doxorubicin–cyclophosphamide group for *BRCA* carriers with early HER2-negative BC (Tung et al., 2020b). The BrightTNess trial demonstrated that the addition of carboplatin increased pCR (Loibl et al., 2018a) and improved event-free survival (Loibl, 2021) compared with paclitaxel alone in unselected TNBC patients. Therefore, for germline *BRCA* mutation carriers with BC treated with neoadjuvant therapy, the routine addition of platinum to anthracycline and taxane-based chemotherapy is not supported. Therefore, we need more evidence to explore the role of platinum salts in early BC with *BRCA* mutations. Several ongoing trials are conducted to investigate the efficacy and safety of neoadjuvant platinum-containing chemotherapy or combined with olaparib in neoadjuvant therapy for operable TNBC (NCT 02978495, NCT 04664972, NCT03150576).

### Platinum Salts and Metastatic Breast Cancer With *BRCA1* Mutations

Germline *BRCA* carriers could benefit from platinum agents in the treatment with metastatic BC (Kriege et al., 2009; Byrski et al., 2012). However, those results should be interpreted with caution due to small sample size. In a phase III trial TNT (Tutt et al., 2018), in the subset of patients with *BRCA*- mutated metastatic BC, patients benefit more from carboplatin versus docetaxel, who yielded a greater objective response rate (ORR) and longer PFS. However, no overall survival benefit was observed. In addition, TNT trial might also be underpowered due to a small sample size for *BRCA1/2* mutation carriers (*n* = 55). In another small-sample-size (N = 11) single-arm trial, 6-mercaptopurine (6 MP) and methotrexate, which could selectively kill *BRCA*-defective cells in a xenograft model, failed to show anti-tumor activity for advanced BC with a *BRCA1* mutation (Roberts et al., 2020).

### Resistance to Platinum-Based Chemotherapy or PARP Inhibition

PARPi-based chemotherapy has shown great promise in clinics; however, not all patients with mutations in *BRCA1* or genes associated with BRCAness will respond to PARPi, as different mutations may have differing effects on the DNA double-stand break repair function and sensitivities to PARP inhibition. There is very limited understanding of what factors may affect PARPi responses in the setting of *BRCA1* mutations and other BRCAness genes. It is also likely that the therapeutic implications may differ in different cancer types, further reinforcing the importance of the context in which BRCA and other HR-related genes function in these malignancies.

In addition, lots of patients acquire PARPi resistance with prolonged PARPi treatment in clinics. Resistance to platinum-based chemotherapy is also a promising predictor for resistance to PARPi, suggesting that they may share a common mechanism. The main molecular mechanisms of PARPi resistance are the cellular availability of the inhibitor, reverse mutations, homologous recombination repair restoration, and restoration of replication fork protection. Firstly, in a murine model of *BRCA1*-deficient breast tumors, tumors with overexpressed drug-efflux transporter genes (Abcb1a and Abcb1b encoding for MDR1/P-gp and Abcg2) showed resistance to PARPi by influencing the cellular availability of the inhibitor. The coadministration of the MDR1 inhibitor could resensitize the tumors to the PARPi (Rottenberg, et al., 2008). Secondly, somatic reversion mutations were found in cfDNA (circulating cell-free) in BC patients who acquired resistance to platinum and/or PARP inhibitors. The *BRCA1* reversion mutation could restore *BRCA1* function(Weigelt, et al., 2017). Thirdly, the fork degradation of deprotected replication forks is mediated by at least three mechanisms, which, upon loss, leads to fork protection and thus to PARPi resistance. (Angelo et al., 2017). Lastly, HR reactivation is dependent on the ubiquitin E3 ligase RNF168 and the loss of 53BP1–RIF1–REV7–Shieldin axis in *BRCA1*-deficient and TP53BP1-deficient cells, leading to PARPi resistance (Nakada et al., 2012; Dev, et al., 2018). In addition, cancer stem cells (CSCs) in *BRCA1* mutant TNBCs were resistant to PARP inhibition, and RAD51 protein levels and activity were elevated. ShRNA downregulated the expression of RAD51 and thereby made CSCs sensitive to PARPi. (Liu et al., 2016). Therefore, RAD51 is a functional biomarker to be used in the clinic to identify PARPi-sensitive cancer patients and select the population who may respond to PARPi therapy (C Cruz, et al., 2018). Moreover, epigenetic modification and restoration of ADPribosylation (PARylation) lead to PARPi resistance as well. Studies show that patients with high probability of resistance to PARPi may obtain a benefit from combinatorial treatment strategies. Since PALB2–BRCA2 recruitment to DNA breaks and RAD51 recruitment to stalled forks are both ATR dependent, the combination of PARPi with ATR inhibitors is expected to overcome PARPi resistance in tumors by restoring HR or restoring fork protection. (Stephanie A Yazinski et al., 2017). It is of great importance to clarify the clinical relevance of the different PARPi resistance mechanisms through more large patient cohorts. These studies will pave the way for patients in the clinic to improve diagnosis, therapy decisions, and outcome.

### Hormone Therapy and Breast Cancer With *BRCA1* Mutations

Carriers of *BRCA1* mutation are more likely to develop TNBC (Atchley et al., 2008). Therefore, for those hormone receptor-positive BC patients with pathogenic *BRCA1* mutations, hormone therapy is essential to delay the progression and prevent the onset of contralateral tumors. Adjuvant tamoxifen (Narod et al., 2000) and aromatase inhibitors (Gutierrez-Barrera et al., 2015) could significantly reduce the risk of contralateral tumors. The addition of contralateral breast irradiation was associated with a significant reduction of subsequent contralateral breast cancers and a delay in their onset (Evron et al., 2019).

### Immunotherapy and Breast Cancer With *BRCA1* Mutations

Immunotherapy is an important approach for cancer treatment, and DNA repair defects are an important factor in enhancing the anti-tumor immune response. The germline *BRCA1* mutation is related to the high mutation burden of TNBC, and the combination of cisplatin and PD-1/CTLA-4 antibody has a more significant tumor inhibition effect than the use of cisplatin alone (Mouw et al., 2017). Therefore, the combination of immune checkpoint inhibitors and chemotherapy can effectively improve the efficacy of HR-deficient tumors. In addition, a number of clinical trials of PARP inhibitors combined with chemotherapy or immunotherapy are under way, and preliminary results show that PARP inhibitors combined with immunotherapy can enhance the killing effect of *BRCA1* germline mutation BC (Mateo et al., 2019; Domchek et al., 2020).

Following the success of previous trials, there is a series of phases two clinical trials currently ongoing with immunotherapy or targeted therapy in BC patients with germline *BRCA1* mutation (Table 2).

**TABLE 2:** Immunotherapy or targeted therapy-based treatment in metastatic BC with *BRCA* mutation: ongoing clinical trials.

Study	Phase	Population	Treatment	Primary endpoint	Status
NCT04053322 DOLAF	II	ER-positive and HER2-negative metastatic or locally advanced breast cancer a germline or somatic brca mutation, or a deleterious alteration of other genes involved in homologous recombination repair (HRR) or in MSI status	Durvalumab plus olaparib plus fulvestrant	PFS	Recruiting
NCT04673448	Ib	Metastatic TNBC with germline BRCA mutation	Niraparib and dostarlimab (TSR-042)	Best objective response	Recruiting
NCT03414684	II	Metastatic TNBC	Carboplatin ± nivolumab	PFS	Active, not recruiting
NCT04584255	II	Early HER2-negative breast cancer with germline BRCA mutation	Niraparib with dostarlimab	pCR	Recruiting
NCT03685331 HOPE	II	BRCA mutation-, hormone receptor-positive, HER2- negative metastatic breast cancer	Olaparib, palbociclib, and fulvestrant	PFS	Recruiting
NCT02203513	II	BRCA1/2 mutation, TNBC	Chk1/2 inhibitor (LY2606368)	ORR	
NCT04556292	II	Locally advanced and/or metastatic breast cancer with BRCA mutation	SC10914	ORR	
NCT03911973	II	BRCA1/2 mutation, TNBC	Gedatolisib (PI3K/mTOR inhibitor) plus talazoparib	ORR	
NCT04240106 LUZERN	II	(HR)+/(HER2)-, MBC with either germline BRCA-mutated or germinal BRCA-wildtype and homologous recombination deficiency	Niraparib + aromatase Inhibitors	CBR	
NCT03931551 OPHELIA	II	HER2-positive BRCA-mutated advanced breast cancer	Olaparib plus trastuzumab	CBR	
NCT04090567	II	Germline BRCA-mutated advanced or metastatic breast cancer	Olaparib with cediranib or AZD6738	ORR	
NCT02849496 (Tung et al., 2020a)	II	HDR-deficient, locally advanced or metastatic non-HER2-positive breast cancer	Olaparib and atezolizumab	PFS	Recruiting

### Beyond *BRCA1* Germline Mutation

In addition to *BRCA1* and *BRCA2* mutations, the BC patients with *BARD1, PALB2, RAD51, FANCA*, *ATM*, *ATR*, and *CHK2* mutations also present an HRR defect and might also share sensitivity to platinum-based drugs and PARPi (Christopher J. Lord and Ashworth, 2016). The above HR genes share the same cancer genetic profile and are called *BRCAness* (Cai, 2020). All these genes’ mutations increase the sensitivity of tumor cells to PARP inhibitors, suggesting that PARP inhibitors may be effective against multiple tumors rather than specific tumors with *BRCA1* mutations. TBCRC 048, a phase II study, emphasized that PARP inhibition is an effective treatment for patients with MBC and germline *PALB2* or somatic *BRCA1/2* mutations (Tung et al., 2020c). Furthermore, in treatment-naïve TNBCs, olaparib monotherapy yielded a high clinical response rate with BRCAness signature (Eikesdal et al., 2020), beyond germline HR mutations (NCT02849496, NCT03990896, NCT04892693). Many similar clinical trials are underway, which will greatly expand the usage of PARP inhibitors.

## Conclusion

*BRCA1* gene is a well-known tumor suppressor gene, and germline *BRCA1* mutation is closely related to the occurrence, development, and treatment of BC. With the advancement and popularization of gene sequencing technology, *BRCA* testing has been widely used in clinical practice. For better outcomes of BC patients with germline *BRCA1* mutation, it is necessary and critical to comprehensively consider the combination of the therapies, and a multidisciplinary consultant is required to make an appropriate individualized management plan. With further exploration on the *BRCA1* gene, more and more patients with *BRCA1* mutations will benefit.

## Abbreviations

CBR, clinical benefit rate; HDR, homologous DNA repair; HER2, human epidermal growth factor receptor 2; HR, hormone receptor; ORR, overall response rate; PFS, progression-free survival; TNBC, triple-negative breast cancer.
